# Elevated Risk of Venous Thromboembolism in People Living with HIV

**DOI:** 10.3390/v14030590

**Published:** 2022-03-12

**Authors:** Quan Zhang, Fei Peng, Meizhi Li, Qiong Yi, Wei Tang, Shangjie Wu

**Affiliations:** 1 Pulmonary and Critical Care Medicine, The Second Xiangya Hospital, Central South University, Changsha 410011, China; zqnichla2007@csu.edu.cn (Q.Z.); 198201030@csu.edu.cn (F.P.); limeizhi@csu.edu.cn (M.L.); 15211366172@163.com (Q.Y.); tangwei1996@csu.edu.cn (W.T.); 2 Hunan Centre for Evidence-Based Medicine, Changsha 410011, China

**Keywords:** HIV, venous thromboembolism, epidemiology, pathophysiology

## Abstract

Human immunodeficiency virus (HIV) has been generally considered as a highly adaptive and rapidly evolving virus. It still constitutes a major public health problem all over the world despite an effective outcome in the prevention and reversal of the development and prognosis by using antiretroviral therapy. The salient question lies in the more frequent emergence of a series of comorbidities along with the prolongation of the life, which deeply affects the survival in such group. Venous thromboembolism (VTE) has been recognized to be the third most common cardiovascular condition within people living with HIV (PWH). In terms of its mechanism of action, the occurrence of VTE is quite multifactorial and complex in HIV. Prior exploration concerning the etiology of VTE in PWH identifies general, disease-specific, and miscellaneous factors for explaining its occurrence and development. VTE has constituted an important role in PWH and may increase its all-cause mortality. Therefore, it is quite necessary to understand VTE from the following aspects of epidemiology, pathophysiology, molecular mechanisms, and therapeutic interventions so as to balance the risks and benefits of anticoagulation and optimize corresponding treatment.

## 1. Background

It has been predicted that there is a relatively higher risk of cardiovascular disease, venous thromboembolic disease, and microvascular disease in people living with HIV (PWH), especially in an aging HIV-infected population. In general, venous thromboembolism (VTE) is a pathological complication and/or triggering event. Its occurrence can be triggered by multiple factors, such as primary or secondary thrombotic abnormalities, chronic diseases, infected pathology in specific patients, and provoking factors, surgery, trauma, catheterization, acute venous stasis, pregnancy, and even some treatments [1].

Thrombotic diseases of PWH develop frequently without common risk factors but show intimate association with HIV infection itself and secondary tumors, opportunistic infection, hypercoagulable state caused by immunosuppression, and vascular endothelial injury. Various previous studies have documented the significance of HIV-specific factors in venous thrombotic event [2,3,4]. In our overview, thrombotic risk factors in PWH are categorized into general factors, disease-specific factors, and miscellaneous factors. In the pathology of infection, several biological mechanisms can theoretically fuel a cause-and-effect relationship between HIV infection and VTE from the following aspects of host response, the virus itself, and even the antiretroviral therapy. The viral replication and antigenic stimulation of HIV stimulates both the host inflammatory and immune response, resulting in the upregulation of various inflammatory cytokines, including CRP, IL-6, IL-8, thymus activation regulated chemokine (TARC), and tissue inhibitor of metalloproteinase-1(TIMP-1). It may also induce monocyte activation, endothelial dysfunction (EDF), and coagulation changes in P-selectin, thrombomodulin, and von Willebrand factor (vWF) [3].

Findings on HIV infection revealed by these studies are of great significance for understanding the relationship between VTE and HIV infection. Effective measures shall be considered for the prevention of thrombosis, especially in the case of severe acute infection after HIV infection. Our overview emphasizes on the possible interaction between HIV infection and the risk of VTE as well as the effect on the management of VTE in PWH.

## 2. Epidemiology

### 2.1. HIV Infection

Acquired immune deficiency syndrome (AIDS), one of the known viral infections, has appeared as a progressive pandemic all over the world since its first report in America in 1981. The Joint United Nations Program on HIV/AIDS (UNAIDS) reported that the numbers of population infected with human immunodeficiency virus (HIV) and diagnosed with AIDS patients reached 38 million in 2020. Despite the application of combination antiretroviral therapy (c-ART) in a large proportion of the infected (25.4 million), there are still 1.7 million newly infected cases and 690 thousand death caused by illness related to HIV/AIDS annually. It poses a great economic and medical threat to the health of human beings all over the world. Significantly, owing to the worldwide use of c-ART for over two decades, dramatic improvements have been realized in the clinical setting in the aspects of the quality and length of life for PWH. Thus, infection with HIV is becoming a common chronic disease since the prolongation of the life of PWH. Such group of patients may experience an increased risk of non-HIV/AIDS causes of end-organ disease, which includes vascular complications, such as VTE, posing new clinical challenges, however.

### 2.2. VTE in PWH

VTE, consisting of deep vein thrombosis (DVT) of the leg or pelvis and pulmonary embolism (PE), is the third most common cardiovascular condition, with an estimated incidence between 0.7 and 1.5 per 1000 person-years [5,6,7]. Besides, VTE shows a varied incidence in populations from different races [8,9,10], which is higher in the Black population, followed by the White population, Hispanics, and Asians/Pacific Islanders (3.8–3.82 vs. 1.54–2.3 vs. 0.67–1.05 vs. 0.63 per 1000 person-years, respectively). It has an average incidence of 1.23 per 1000 person-years in United States, 1.31 per 1000 person-years in Europe, and approximately 15–20% of the levels recorded of this in Asia (0.175–0.63 per 1000 person-years) [11,12,13]. Meanwhile, the incidence of VTE varies by age, which increases more exponentially in adults for both males and females than children under 18. It may be explained by the rapidly aging population and higher prevalence of cardiovascular disease and cancer. However, most studies demonstrated the incidence of women during childbearing ages of 20–40 is higher than that in men [14,15], and the over 13 million enrollees from the USA for the 5-year pre-pandemic period (2015–2019) indicates that VTE incidence tended to increase with age and was higher for women under 55 [16]. However, VTE can be prevented in clinical practice, and it is of great significance to screen high-risk population for VTE. More interestingly, patients with HIV may also experience higher risk of VTE, which may be considered as an independent risk factor for developing VTE. Prior epidemiologic studies reported a 2- to 10-fold increased risk of first VTE in PWH when compared with age-matched controls [17,18,19]. As listed in Table 1, the studies vary greatly in sample size and quality and reported frequencies ranging from 0.52 to 10.1 per 1000 person-years [2,17,18,20,21,22,23,24,25,26,27,28,29,30,31,32]. Even in the same country, within overlapping time periods, the VTE incidence rate is quite different; it seems that the smaller studies with observations have a relatively higher rate, indicating that the deviation is larger, and the incidence rate has not been adjusted or standardized, or even by doing this, the small sample size still cannot really reflect the local demography. Secondly, some studies just collected the inpatients and only calculated the incidence of DVT [23], but some enrolled both the outpatients and inpatients and calculated the incidence of DVT, PE, multi-site venous thrombosis [18], and even arterial thrombo-embolic events [32].

PWH might also experience a relatively higher risk of VTE in the case of a lower plasma CD4+ T-cell counts or/and higher viral load as well as current opportunistic infections in those populations [20,28,33]. Additionally, patients with mono-infection of HIV might be at higher risk for PE-related hospitalizations than those with co-infection of HIV and hepatitis C virus (HCV), of which there was a significant increase in the incidence of HIV/HCV co-infection from 2008 to 2013 [34]. Furthermore, there is an increase in mortality associated with patients experiencing VTE when compared to patients without VTE. The adjusted 30-day all-cause mortality rate was 5.1–6% and 9.1–12% among Medicare beneficiaries with DVT and PE, respectively, with the 1-year mortality of 10% on average [35,36]. In 2004, Fultz et al. [24] revealed that thrombosis was significantly associated with poorer survival in both HIV-positive patients (hazard ratio (HR) = 2.00; 95% CI: 1.71 to 2.34) and the HIV-negative controls (HR = 2.15; 95% CI: 1.62 to 2.86). In the United States, 375,000 to 425,000 new cases are assumed annually, and the overall cost is estimated at USD 7–10 billion annually, which is a heavy burden [37].

## 3. Pathogenic Factors of HIV-Associated Thrombosis

Generally, blood coagulation, which can be activated by biological procoagulant mechanisms expressed by HIV infection, together with multiple clinical factors contribute to the overall thrombotic risk of these patients significantly. Considering the involvement of various risks among PWH, our review elaborates on different aspects of risk factors and specific pathophysiology, which render the pathogenesis of HIV-associated thrombosis unique.

### 3.1. General Factors

**Figure 1:**
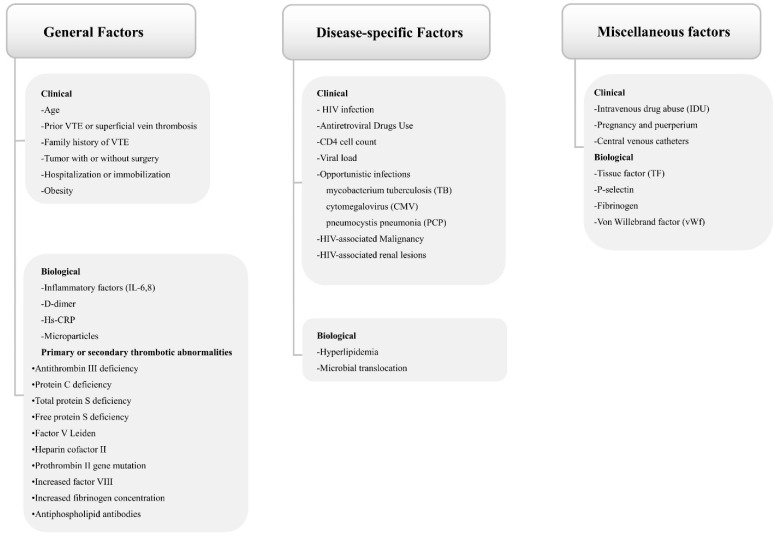
Various factors implicated in HIV-associated thrombosis.

As shown in Figure 1, general clinical factors and biological factors both may produce a risk of thrombosis in the general population with/without HIV/AIDS. Listed general clinical factors include age, prior VTE, tumor with/without surgery, hospitalization, immobilization, obesity. It should be noted that not all of them are demonstrated in all PWH. Increasing age is dramatically associated with the incidence VTE, and it has been reported in various studies that the elderly may have an evidently higher possibility to develop VTE than that the young [5,38]. Currently, VTE incidence for patients under the age of 18 years is estimated to be roughly 0.01–0.02 per 1000 person-years [39]. In a Korean study, which included 3611 children with cancer over a 15 year period, only 0.9% developed VTE [40]. In accordance with the data of adult incidence, age is the strongest risk factor for VTE, showing the incidence of DVT per 1000 person-years increases from 0.08 for those age 25–29, to 0.39 for age 35–39, 0.82 for age 45–49, 0.91 for age 55–59, 1.13 for age 65–69, and 2.94 for age 75–79. Subjects aged over 45 years may have a significantly higher risk of VTE, approaching 5–6 per 1000 person-years by age of 80 [5,41]. It can be explained by the decreased mobility and other illnesses predisposing to thrombosis in the elderly. While factors such as improved awareness of VTE diagnoses and recent developed diagnostic techniques or a combination of these factors may contribute to a higher incidence of VTE, PWH have a median age of 40 when developing VTE, which is generally 20 years younger than the non-infected [22].

On the other hand, biologically, the risk of VTE can be stimulated by abnormal blood components, such as platelet activation, elevated D-dimer levels, tissue factor (TF), fibrinogen, vWF, and P-selectin. As supported by one previous research, after the withdrawal of HIV treatment, there was a negative correlation of D-dimer, HIV-1 RNA levels, and platelet counts, suggesting that platelets and coagulation factors might be activated by HIV-1 viremia [42]. The inherited thrombophilia contains high levels of antiphospholipid-anticardiolipin antibodies, serum homocysteine, lupus anticoagulant, and plasma factor VII activity but low levels of antithrombin III-activated protein C (PC) resistance, PC and protein S(PS) deficiencies, heparin co-factor II, and antithrombin deficiency.

### 3.2. Disease-Specific Factors

In addition to general risk factors, HIV-specific factors are also influential in increasing the risk of VTE disorders. The clinical and biological pathogenic factors within the proposed category are exclusive to this unique disease (Figure 1).

#### 3.2.1. Viral Infection

Without doubt, the virus-related risk factors constitute the main body of the clinical factors. In the prior Strategies for Management of Antiretroviral Therapy (SMART) study, D-dimer and as well as IL-6 and high-sensitive-CRP exhibited significant predictive effect on patients’ mortality [43]. Moreover, considering the role of HIV infection in platelet activation, the activation of both the inflammatory and coagulation systems may be stimulated by HIV infection, consequently to the development of venous thrombotic events. The hemostatic balance may be disordered due to the dysfunction of pro- and anti-coagulant factors.

#### 3.2.2. Antiretroviral Drugs Use

Typically, c-ART is performed by using two nucleoside reverse transcriptase inhibitors (NRTIs) and one non-nucleoside reverse transcriptase inhibitor (NNRTI), a protease inhibitor (PI), or integrase inhibitor (II). It has been recognized to be the gold standard for treating HIV and can prolong the length of lifetime for the majority of PWH as evidenced by the extensive application of the proposed therapy. However, and inevitably, long-term use of antiretroviral drugs may result in gradual increase in the side effects of the treated patients, which is speculated to be related to the effect of certain components of ART on the risk factors. To be specific, the use of PIs may cause metabolic changes (e.g., hyperlipidemia), predominantly an increase in circulating low-density lipoprotein and cholesterol levels [44]. Studies found that some c-ART regimens, PIs in particular, may aggravate embolism during the application of HAART [21,45,46,47,48] and irritate EC junctions as well as actin cytoskeleton in ECs to promote endothelial damage [49], whereas the significance of c-ART still remains controversial in the clinical setting. For instance, additional studies revealed weak or even no meaningful relationship between VTEs and ART [18,28,50]. Especially, the advancement of antivirus drugs in the combination therapies contributes to better virologic outcomes and minimal adverse events.

#### 3.2.3. CD4 Cell Count and Viral Load

At present, scholars have nearly reached a consensus that active HIV viral load and CD4 cell counts have a clear association with the progression of VTE among PWH despite the absence of consistent results in few studies [51]. According to the current retrospective cohort studies [17,18,20,52], as independent risk factors, CD4 counts <200 cells/µL and viral load >100,000 copies/mL exhibited positive association with the risk of a VTE. Specifically, the earlier the time of HIV diagnosis, the higher the overall effect of CD4 cell count was on the risk of a venous thrombotic event, while a negative association was found in those with prolonged HIV duration [52], which was associated with the prolonged duration of HIV viremia and/or delay in ART initiation and the nadir CD4 cell count as well [53]. The association between low CD4 cell counts and VTE have been reported to be correlated with an increasing hypercoagulable state, which was found within progressive immune suppression during HIV infection as well as high HIV viral loads [20,26]. For example, subjects with lower CD4 cell counts showed a higher risk of developing PS and PC deficiencies [54,55]. Moreover, the risk of VTE has a stronger association with intravenous drug use or HAART drug although it may be related to CD4 cell count and viral load as suggested in our overview. Moreover, the risk of VTE in PWH who have been well treated by HAART and who maintain normalized CD4 cell counts (>500cell per µL) was almost close to that in general population.

#### 3.2.4. Opportunistic Infections

PWH is an immune deficiency disease, with death caused by opportunistic infection primarily. ART can induce the occurrence for most opportunistic infections generally, which, however, has a better short-term therapeutic outcome in the first year of treatment [56]. It is not difficult to understand that patients with opportunistic infections may develop VTE easily by lowering PS levels and through immobility, which has been confirmed by clinical studies [20,57,58]. Thrombotic events are most commonly reported with mycobacterium tuberculosis (TB), cytomegalovirus (CMV), and pneumocystis pneumonia (PCP).

The reported data globally showed that approximately 10 million people developed TB, which occupied the most part of opportunistic infection in patients with HIV infection; besides, the incidence of TB was 2.1% (range, 1.9–2.4%) per 100 person-years with HIV. The risk of developing TB among PWH was 18 (range, 15–21) times higher than in the rest of the global population [57]. Furthermore, the presence of TB has been commonly reported during a VTE in PWH [58]. Once acting as the most universal virus co-infected with HIV infection [59], CMV is now much less frequent due to the suppressive combination of ART [60,61,62]. In a recent large cohort study, 56 cases with CMV infection out of 32,198 HIV patient-years of follow-up care were identified during 2004–2015. The corresponding incidence rate was 1.7 cases per 1000 patient-years, with a median follow up of 4.5 years [18]. Furthermore, previous animal and clinical studies have suggested the potential role of CMV in atherosclerosis; in addition, case-control studies found the prevalence of seropositivity for CMV was higher than that of VTE in general patients [63,64,65,66], and accordingly, the proposed association is not unique to individuals with HIV infection [67]. As for the reasons, both of the active viral co-infections are associated with inflammation, aging, and procoagulant state; besides, several mechanisms have been proposed to explain CMV-induced vascular thrombosis [61]. In terms of its mechanism theoretically, the infectious state can transiently increase the risk of thrombus formation, which can be manifested in the enhanced platelet and leukocyte adhesion to the endothelium, factor X activation, smooth muscle proliferation, and increased production of thrombogenic factors [68]. It is still the most common and serious opportunistic respiratory infection in patients with AIDS. However, there is a substantial decline in the incidence of infection in this population owing to the widespread use of prophylaxis and ART. There are a few but not many reports of the presence of VTE in HIV-co-infected with active PCP, which may be the result of co-infection causing hypercoagulable state [69,70].

#### 3.2.5. HIV-Associated Malignancy

Cancer patients may experience an approximately 4–7-fold (4% to 20%) increased risk of VTE [71,72]. Cancers are frequent among PWH, such as AIDS-defining cancers (Kaposi’s sarcoma, non-Hodgkin lymphoma, invasive cervical cancer), infection-related malignancies (Hodgkin lymphoma, liver cancer, anal and oral cavity cancer), and non-AIDS-defining cancers (lung cancer) [73]. Meanwhile, the proportion of complicated malignancy(13/30 [51]), 13/45 [23], 21/232 [18])has been reported in VTE patients complicated with HIV-infection.

#### 3.2.6. HIV-Associated Renal Lesions

Whether chronic kidney disease or acute kidney injury (AKI), patients with renal insufficiency commonly have coagulation disorder [74,75]. It has been revealed that compared with patients with normal renal function, there were faster coagulation speed and increased clot strength in patients with chronic renal insufficiency complicated with cerebral hemorrhage, suggesting a relatively higher state of hyper-coagulation in the latter group [76]. Meanwhile, as suggested by Huang MJ et al. [77], there was an evidence of more severe hyper-coagulation in nephrotic syndrome patients with AKI. It may be attributed to the active immune inflammation of AKI that can be involved in activating the coagulation system. Indeed, ART contributes a great deal to changing the incidence and spectrum of HIV-associated kidney diseases. The current prevalence of HIV-associated nephropathy (HIVAN) is approximately 20% among HIV-infected patients [78]. Therefore, there is a reason to believe that HIV-associated renal lesion can be attributed to the following hematological abnormalities: fibrinogen and coagulation factor VIII, vWF, and deficiency of antithrombin III, PC, and PS [79,80]. Furthermore, anticoagulants, such as warfarin, can in turn cause renal function damage. According to previous studies, about 20.5% of warfarin-treated patients developed at least one episode of warfarin-related nephropathy during treatment [81,82].

#### 3.2.7. Hyperlipidemia

Despite the presence of dispute in the relationship between hyperlipidemia and VTE, accumulated epidemiological evidence supports their possible link [83,84,85] and the value of statins in reducing the risk of VTE [86]. One previous meta-analysis [87] revealed that subjects with VTE had significantly higher mean total cholesterol (TC) and triglycerides (TG) concentrations and lower mean high-density lipoprotein cholesterol (HDL-C) concentrations than those without VTE. The use of ART, especially PI, has an unfavorable effect on the lipid profile, glucose metabolism, and changes in body fat deposition, at least during early treatment earlier regimens’ treatment [88]. For instance, before the era of C-ART from 1996, Grunfeld et al. [89] found that relative to decreased trends of TC and HDL-C in HIV-infected patients, there were elevated plasma TG and free fatty acid levels in patients with AIDS. However, the use of PI-based treatment resulted in the increase of TC and TG levels [90,91]. In addition, previous observational research revealed a lower incidence of inducing metabolic abnormalities following the application of NNRTIs and NRTIs compared to the use of PIs [92]. The authors argued the mechanisms that dyslipidemia was correlated with the lipodystrophy, insulin resistance, and increase in fasting glucose as well [93]. As for the possible reason, the reconstitution of ART may promote the release of inflammatory mediators, which may further induce both metabolic syndrome and immune reconstitution inflammatory syndrome (IRIS) [94]. A lower CD4 count may result in poorer immunity and enhanced abnormal fat distribution, namely central adiposity and loss of peripheral fat [95].

#### 3.2.8. Microbial Translocation (MT)

MT is such an in vivo movement of microbial products from the gut mucosa into circulation. It occurs in subjects with impaired tight epithelial barrier and mucosal immune dysfunction [96,97]. MT has been proposed as a major driver of chronic immune activation and inflammation in PWH, which occurs when microbial products traverse the tight epithelial barrier of the gastrointestinal tract [98,99]. Authors discovered that there was an elevation in LPS in untreated HIV-infected individuals, and MT correlated negatively with platelet aggregation [100]. Simultaneously, it has been reported that both LPS and flagellin can increase the expression of TF and activate the coagulation cascade [101].

### 3.3. Miscellaneous Factors

According to prior research, as many as 47.6% of intravenous drug abusers (IDUs) were reported as having suffered from DVT, and this group of population constitutes approximately half of patients aged less than 40 years old with DVT. The overall morbidity of HIV were 12.55% and 1.05% in IDUs and non-IDUs, respectively [102]; besides, in the presence of HIV infection, IDU patients may have a higher risk of VTE (15-fold) than that of subjects without IDU [17]. Relevant critical pathophysiological factors may consist of vein lesions caused by multiple vein punctures and no sterility injections, type of drugs injected intravenously (heroin, cocaine, amphetamine, opioids), injection of insoluble medicinal particles, and irritation of vein walls by adulterants resulting in vein hardening, infection, and inflammation [103]. Moreover, the intravenous injection of the aforementioned drugs may increase fibrinogen content, aggregate red blood cells and platelets, and induce their dysfunction.

Finally, tissue factor (TF), fibrinogen, P-selectin, pregnancy, puerperium, and central venous catheters exert a prothrombotic effect. Increased frequencies of TF expression in activated monocytes may be initiated by elevated inflammation in PWH and have been associated with higher HIV-RNA levels [101,104]. In contrast, expression of TF on the surface of activated monocytes and TF-positive microparticles may stimulate thrombosis and thus increase the risk of clotting, inflammation, and atherosclerosis [105]. Moreover, fibrinogen, P-selectin, pregnancy, and puerperium as well as the presence of central venous catheters may play a more direct role in the pathogenesis of VTE in PWH.

## 4. Pathophysiology

Acting as a complex pathology, VTE refers to the formation of intravascular blood clots in the venous circulation. Stasis of blood flow, vascular damage, and hypercoagulability of blood have been hypothesized to be the major factors responsible for the formation of clots [106,107]. The pathogenesis VTE may be promoted by one or a combination of these factors in general. In some cases, however, no causal factor can be identified. The venous valve is regarded to be the initial onset site for VTE. Both inherited and acquired factors may contribute to this disruption. A common opinion is that it is multifactorial and controversial concerning the pathophysiology of VTE in PWH. However, the particularity of VTE in PWH lies in that the VTE of PWH often occur without these common risk factors identified previously. It shall be noted that HIV infection itself manifests in the continuous endothelial dysfunctional immune activation and inflammation, hence showing increased risk of thrombosis. There exists association between infection, secondary tumors related/non-related to HIV, antiretroviral therapy, and thrombosis via endothelial activation, coagulation, and natural anticoagulation. These phenomena persist during ART despite modest improvements.

### 4.1. Immune Activation and Inflammation

Chronic inflammation is a common phenomenon in subjects with HIV infection. Of course, there may be certain chronic infections that are less understood. Patients may show sustained systemic inflammation, acting as hallmark of HIV infection at the early stage weeks of infection [108]. A growing body of evidences suggests inflammation has been accepted as a common pathway involved in the development of VTE triggered by various risk factors, which also plays a key role in the pathophysiology of this disorder [109]. A feasible mechanism is that thrombus formation may be induced in an intact vein owing to the inflammation of the vessel wall, among which inflammation and coagulation systems may be activated simultaneously through a common pathway. Generally, the role of inflammation in mediating thrombotic responses is realized by the procoagulant activation, anticoagulant suppression, and fibrinolysis inhibition, in other words, by enhancing the hypercoagulable state and aggravating endothelial damage [110]. As a matter of fact, there is a mutual cause-and-effect relationship between coagulation and inflammation. It has been shown that the release of ultra-large adhesive strings of vWF from activated ECs play a core role for thrombo-inflammation, which is in response to inflammatory mediators, enhancing the exposition of TF by ECs and monocytes/macrophages [111].

### 4.2. Hypercoagulable State

Infection with HIV is a type of hyper-coagulation accompanied by the risk of developing thromboembolic disorders, which are serious and potentially life-threatening. A hypercoagulable state can be considered as a condition that is characterized by increased/activated procoagulant components or decreased/inactivated anticoagulant factors. It can hence lead to a disordered hematological system that is prone to thromboembolism. Whether acute or chronic, HIV infection can be developed from both the direct action of pathogens itself and its effect on the immune system. As it is known to all, HIV replication without treatment may result in decreased coagulation factors (or lack of increases), which are dependent on hepatocyte function. Meanwhile, the replication of this virus may induce complex alterations in the extrinsic pathway coagulation factors and increase of thrombin generation [112]. According to a study carried out by Funderberg et al. [101], there was an increase in TF expression in monocyte along with HIV infection and exhibited a correlation with HIV viral load directly as well as D-dimer levels and inflammatory marker sCD14. Correspondingly, the described abnormalities can reflect the severity of HIV-associated immunosuppression (CD4 cell counts) and with the existence of concurrent infectious or neoplastic diseases to some extent.

In addition, platelet activation is also another potential mechanism for the causes of hypercoagulable state. To be specific, chemokine CXCL4 release can be induced after activating platelets, which exert an inhibitory effect on HIV infection at the stage of viral entry. Purinergic products (ATP and ADP) secreted by platelets is a critical link during the activation of platelet in the presence of HIV infection [113]. Meanwhile, with the activation of platelets, it is possible to activate coagulation factors and mediate leucocyte function. Moreover, the activated leucocytes can also be involved in the reciprocal regulation of platelet function. It was once reported that gp120, acting as the HIV envelope protein, can promote the expression of TF to initiate coagulation cascade through the activation of human arterial smooth muscle cells. Significantly, gp120-induced activation of ASMCs functions critically in the prothrombotic phenotype of the PWH [114]. In this regard, the hypercoagulable state correlated with HIV infection and other chronic infections must be considered as a permanent risk factor for VTE [115].

### 4.3. Endothelial Dysfunction (EDF)

Endothelium, or ECs, is a monolayer of cells where blood vessels line, which can play an anticoagulant role. Rather than endothelium-tropic, HIV-1 is actually an T-cell tropic virus, and it is still a controversial issue concerning the ability of HIV to infect ECs directly. The tissue source and function of ECs may determine the infectious status of HIV-1 to infect ECs. The permeability of endothelium may be improved following the upregulation of cellular adhesion molecules and procoagulant factors caused by internal pro-inflammatory stimuli or external trauma [116]. As described above, HIV infection can aggravate systemic inflammation and coagulation. Importantly, these two disorders may be secondary to aggravated migration of monocytes across the endothelium and forming foam cells to induce EDF [117]. Indeed, there may be the presence of endothelial activation and damage in PWH as indicated by the abnormality in levels of common inflammatory and coagulation markers involving soluble intercellular adhesion molecule and soluble vascular cell adhesion molecule in addition to hCPR, IL-6,TNF-α,D-dimer, etc. [118,119].

**Figure 2:**
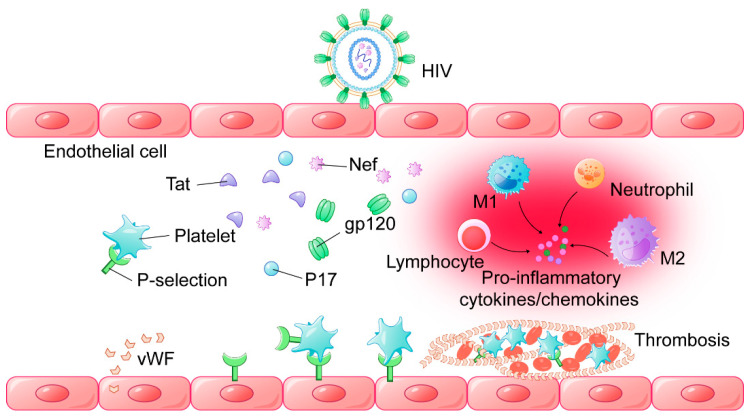
Mechanisms of endothelial activation in the condition of HIV infection. HIV-1-encoded proteins (Tat, Nef, gp120, P17) contribute to the synthesis and release of different cytokines/chemokines from platelet activation (connected with p-selectin), monocytes M1 and M2, neutrophils, and lymphocytes, driving inflammation activation and adhesion of vWF with platelets in the tube wall by linear distribution, which can trap leukocytes and erythrocytes, promoting thrombosis formation.

EDF is a state of aberrant endothelial cell activation, which is also a possible inducer of thrombosis [120]. Besides arterial atherosclerosis, EDF also relates to the prothrombotic state throughout the vasculature in a broader context [121]. It has been proposed that HIV-1 proteins and antiretroviral drugs may induce endothelial damage by many ways. Monocytes infiltrating into the vascular intima are divided into different kinds of macrophages: the M1 subset to promote inflammation and the M2 subset, which is inflammatory resolving cells. HIV-1 may disturb the balance between M1/M2 ratio to stimulate EDF via cytokine perturbation [122]. In particular, protein Tat of HIV-1 could enhance angiogenesis, migration, apoptosis, inflammation, as well as monocyte chemoattractant protein-1 (MCP-1) and IL-6 release, which are linked to enhanced ROS production, increased EC permeability, and adhesion molecule expression [123]. Meanwhile, protein Nef of HIV could also promote EDF via apoptosis of ECs through an NADPH oxidase-dependent mechanism and MCP-1 production by regulating the activation of NF-κB signaling pathway [124]. It may further result in cell death, apoptosis, MCP-1 release and inflammation, and alter cholesterol homeostasis. Gp120 can foster cell death, apoptosis, and endothelin-1 (ET-1) secretion, while HIV-1 matrix protein p17 triggers inflammation, angiogenic molecules, monocyte migration, lymphangiogenesis, ET-1 and MCP-1 release, indicating additional and indirect roles of p17 in causing EDF [125] (Figure 2).

Moreover, a few antiretroviral drugs, particularly PIs, can cause alterations of plasma lipoprotein metabolism, thus increasing the risk for EDF [126]. In addition, high HIV-1 RNA levels in plasma were also revealed to be related to EDF, a well-established predictor of atherosclerosis.

## 5. Antithrombotic Agents in PWH

The 2020 ASH guidelines on antithrombotic and thrombolytic therapy do not mention HIV-infected patients. However, it is necessary for us to seriously put forward the potential interaction role of antithrombotic agents in PWH, for the drug–drug interactions (DDIs) between ART and other medications have been widely described. The ARV drugs, especially PIs, have variable effects on CYP450 liver enzymes that metabolize warfarin. Warfarin DDIs are variable, some causing an increase in levels and some a decrease; therefore, close monitoring of the INR is recommended. It is demonstrated that patients on efavirenz-based regimens require lower weekly warfarin doses than patients on lopinavir/ritonavir regimens [127], whereas PWH being treated for tuberculosis need a higher dose of warfarin [128]. Inhibition of CYP 450 enzyme by PIs or pharmacological promoters may lead to an increase in Direct Oral Anticoagulants (DOACs) concentration and potentially increase the risk of bleeding. The induction of CYP450 enzyme by NNRTIs may reduce the concentration of DOACs, resulting in treatment failure [129]. Management of these interactions should involve careful selection based on patient characteristics; ART and anticoagulants with a low potential for DDI should be chosen.

## 6. Conclusions

There is an absence of sufficient clinical attention of HIV-associated thrombosis, and even the prevalence of thrombi embolic events differ within different populations with different ethnic and genetic backgrounds living in different circumstances. In addition to enhancing the understanding of its pathogenesis, it may be helpful to offer novel therapeutic targets of HIV-associated thrombosis. Finally, biomarkers predicting the activation of the clotting system can be used to screen high-risk groups of VTE and to develop anticoagulation strategies to prevent thrombosis in HIV patients more accurately and intentionally. In addition, multidisciplinary cooperation of cardiologists, infectologists, and hematologists will also help to elucidate HIV-associated VTE clinically and pathophysiologically and to promote the development of a personalized scheme of anticoagulation in targeted patients in the future.

## Figures and Tables

**Table 1:** VTE incidence in patients with HIV infection in different countries.

Author	Years Studied	Nation	Population Size (VTE/HIV)	Incidence × 1000 Person-Years
Saif [20]	1993–1998	USA	10/131	7.6
Sullivan [21]	1990–1998	USA	273/103,263	2.6
Copur [22]	1998–1999	USA	10/362	2.7
Saber [23]	1995–2000	USA	45/4752	0.95
Fulz [24]	1996–2001	USA	480/29,000	5.7 ^adjusted^
Erbe [25]	1998–1999	Germany	3/49	6.12
Ahonkhai [26]	1989–2004	USA	160/-	5.4 ^adjusted^
Malek [27]	1996–2004	USA	6944/131,2956	0.52
Crum Cianflone [28]	1996–2007	USA	17/465	3.7
Rasmussen [17]	1995–2007	Denmark	-/4333	3.2
Willem M. Lijfering [29]	2006	Netherland	11/109	10.1
Durand [30]	1996–2011	Canada	87/4424	3.59 ^adjusted^
Musselwhite [2]	1995–2010	USA	23/2072	1.11
Tarus [31]	2009–2012	Kenya	11/200	5.5
van den Dries LW [32]	2003–2013	Netherland	37/1679	2.21
Howard JFB [18]	2003–2015	Netherland	232/14,389	2.5 ^adjusted^

Note. - indicates unknown population or VTE events in the study.

## Data Availability

Not applicable.
